# Randomized controlled study to evaluate the efficacy and safety of soticlestat as adjunctive therapy in adults with complex regional pain syndrome

**DOI:** 10.1093/pm/pnac198

**Published:** 2022-12-20

**Authors:** Stuart Ratcliffe, Dimitrios Arkilo, Mahnaz Asgharnejad, Sudipta Bhattacharya, R Norman Harden

**Affiliations:** St Pancras Clinical Research, London, EC2Y 8EA, United Kingdom; Takeda Pharmaceutical Company Limited, Cambridge, MA 02139, United States; Takeda Pharmaceutical Company Limited, Cambridge, MA 02139, United States; Takeda Pharmaceutical Company Limited, Cambridge, MA 02139, United States; Departments of Physical Medicine and Rehabilitation and Physical Therapy and Human Movement Science, , Northwestern University Feinberg School of Medicine, Chicago, IL 60611, United States

**Keywords:** complex regional pain syndrome, CRPS, interdisciplinary treatment, small sample size

## Abstract

**Objective:**

The objective was to investigate the efficacy and safety of soticlestat as adjunctive therapy in participants with complex regional pain syndrome (CRPS).

**Design:**

A proof-of-concept phase 2a study, comprising a 15-week randomized, double-blind, placebo-controlled, parallel-group study (part A), and an optional 14-week open-label extension (part B).

**Methods:**

Twenty-four participants (median age 44.5 years [range, 18–62 years]; 70.8% female) with chronic CRPS were randomized (2:1) to receive oral soticlestat or placebo. Soticlestat dosing started at 100 mg twice daily and was titrated up to 300 mg twice daily. In part B, soticlestat dosing started at 200 mg twice daily and was titrated up or down at the investigator’s discretion. Pain intensity scores using the 11-point Numeric Pain Scale (NPS) were collected daily. The Patient-Reported Outcomes Measurement Information System (PROMIS)-29, Patients’ Global Impression of Change (PGI-C), and CRPS Severity Score (CSS) were completed at screening and weeks 15 and 29.

**Results:**

From baseline to week 15, soticlestat treatment was associated with a mean change in 24-hour pain intensity NPS score (95% confidence interval) of –0.75 (–1.55, 0.05) vs –0.41 (–1.41, 0.59) in the placebo group, resulting in a non-significant placebo-adjusted difference of –0.34 (–1.55, 0.88; *P *=* *.570). Statistically non-significant numerical changes were observed for the PROMIS-29, PGI-C, and CSS at weeks 15 and 29.

**Conclusions:**

Adjunctive soticlestat treatment did not significantly reduce pain intensity in participants with chronic CRPS.

## Introduction

Complex regional pain syndrome (CRPS) is a rare pain condition with an estimated incidence of 26.2 per 100,000 person-years [1, 2], and women are two to four times more likely to be affected than men [1, 3, 4]. CRPS typically develops 4–6 weeks after direct trauma and involves sensory, vasomotor, sudomotor, trophic, and/or motor abnormalities [5]. Severely affected individuals experience chronic pain and multiple system dysfunctions, which are associated with substantial impairment to daily functioning and degradation of quality of life (QoL) [4, 6, 7].

Given the multifaceted nature of CRPS, an integrated multidisciplinary treatment approach is recommended [2, 8]. Pharmacotherapy and/or interventional procedures are often recommended to manage specific signs and symptoms, together with appropriate physical therapy and psychological interventions to help to ameliorate symptoms [9]. However, the condition remains difficult to treat and, with few treatments formally tested in randomized controlled trials, an evidence‐based approach to managing CRPS is difficult [2, 8, 10].

The pathophysiology of CRPS is not well understood, but it is thought to be a neurological disorder with prominent features of both central and peripheral sensitization [11, 12]. Key pathways include nociceptive sensitization, immune/inflammatory activation, vasomotor dysfunction, and maladaptive changes throughout the nervous system [12]. Central sensitization is mediated in part by activation and upregulation of glutamate receptors, potentially leading to chronic pain, hyperalgesia, allodynia, and perhaps propagation of pain to adjacent non-injured areas [12]. In support of this mechanism, the N-methyl-D-aspartate (NMDA) receptor antagonist ketamine may provide effective short-term pain relief in a subset of patients with CRPS [13, 14]. However, evidence supporting the long-term efficacy of ketamine treatment is lacking [15].

Soticlestat (TAK-935) is a first-in-class selective inhibitor of cholesterol 24-hydroxylase (CH24H; also known as CYP46A1) [16, 17]. CH24H converts brain cholesterol to 24S-hydroxycholesterol (24HC) to maintain cholesterol homeostasis in the brain [16, 17]. Given the involvement of 24HC in positive allosteric modulation of the NMDA receptor [18] and induction of inflammatory gene expression in human neural cells [19], 24HC may also play a role in central nervous system disorders such as CRPS. In preclinical studies, soticlestat lowered brain 24HC in a dose-dependent manner in wild-type mice [16]. Soticlestat was also investigated in a chronic post-ischemic pain (CPIP) model, which has been shown to mimic several key features of CRPS such as distal limb inflammation and allodynia [20]. From 6 hours post ischemia to day 7, CPIP mice treated with soticlestat showed a significant increase in paw withdrawal threshold compared with controls (data on file). The pharmacodynamic activity of soticlestat in humans has been demonstrated in a multiple-rising-dose phase 1 study in healthy volunteers, in which soticlestat dose-dependently reduced plasma 24HC levels [21].

Here we report findings from a pilot proof-of-concept phase 2a study, designed to examine the preliminary efficacy and safety of soticlestat as adjunctive therapy in participants with CRPS (ClinicalTrials.gov identifier: NCT03990649).

## Methods

### Study design

**Figure 1.**
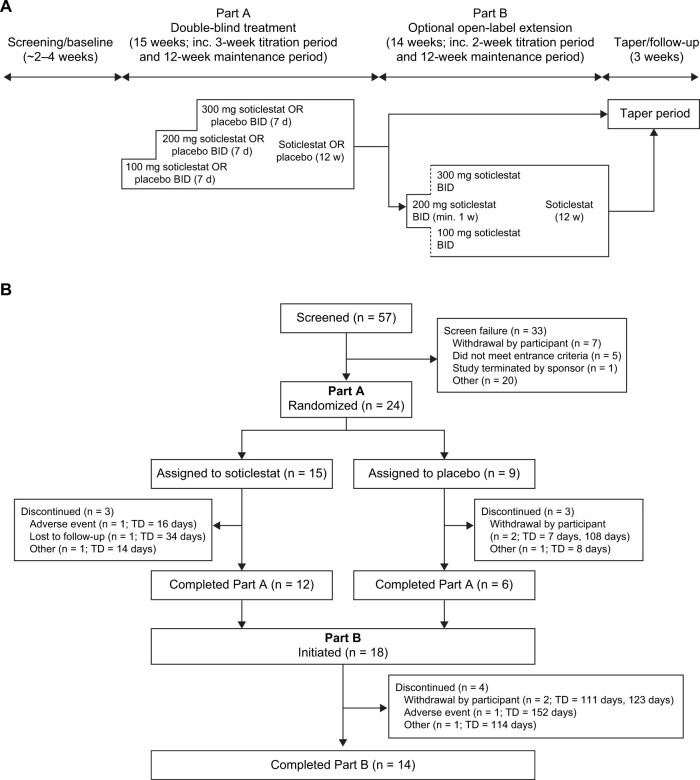
(**A**) Study schematic and (**B**) study flowchart. BID = twice daily; TD = treatment duration.

This phase 2a study was conducted at three sites in the United Kingdom, with two sites randomizing patients (one site recruited only one patient, who did not subsequently pass screening). The study comprised two parts (Figure 1A): part A was a randomized, double-blind, placebo-controlled, parallel-group study consisting of a screening period, a 3-week titration period, a 12-week maintenance period, and a taper period and/or follow-up (for those not continuing to part B). For part A, participants, caregivers, investigators, and outcomes assessors were blinded to treatment assignment. Part B was an optional open-label extension study for those who completed part A, and comprised a 2-week titration period, a 12-week open-label maintenance period, and a taper period and/or follow-up.

The study was conducted according to the ethical principles of the Declaration of Helsinki and the International Council for Harmonisation Harmonized Tripartite Guideline for Good Clinical Practice. All participants provided written informed consent and the study protocol was approved by the institutional review board of all study sites before commencement of the study.

### Participants

Eligible participants were aged 18–75 years with chronic (≥ 6 months since onset of symptoms) CRPS according to the Budapest Criteria, as judged by the investigator at the screening visit [5]. Other key eligibility criteria included: an average 24-hour pain intensity 11-point Numeric Pain Scale (NPS) score of ≥ 4 and ≤ 9 (calculated by averaging the daily 24-hour pain intensity scores for the 7 days before randomization); a history of failure of one or more standard-of-care therapies for the treatment of CRPS as judged by the investigator; and stable use of pain medications and non-drug treatments for 1 month before screening and throughout part A.

Individuals who met any of the following criteria were not eligible for study entry: current or planned use of ketamine, or use (intravenous or oral) within the previous 6 weeks; unstable or clinically significant neurological, psychiatric, cardiovascular, or another condition that might have an impact on their ability to participate in the study or potentially confound the study results; history of convulsions or an increased risk of convulsions; and any history of alcohol, opioid, or moderate-to-severe substance use disorder.

### Treatment

During part A, participants were randomly (2:1) assigned to receive oral soticlestat or placebo (Figure 1A). Soticlestat dosing started at 100 mg twice daily for 7 days and was titrated up to 300 mg twice daily before the 12-week maintenance period. During part B, soticlestat dosing started at 200 mg twice daily, and could be decreased to 100 mg twice daily or increased to 300 mg twice daily at the discretion of the investigator before the 12-week maintenance period.

Dose selection was based on a comprehensive analysis of the safety, tolerability, pharmacokinetic, and pharmacodynamic data from single- and multiple-dose phase 1 studies in healthy volunteers [21, 22], as well as the projected pharmacologically active doses in humans from mice.

### Outcomes

The primary efficacy endpoint was change in mean 24-hour pain intensity NPS score (mean of three measurements collected during 1 day by electronic pain diary [E-diary]) from baseline to week 15 (end of part A). Secondary efficacy endpoints included the following: percentage change in mean 24-hour pain intensity NPS score from baseline to week 15; percentage of responders (defined as showing ≥ 30% improvement in the 24-hour pain intensity NPS score) at week 15; change and percentage change from baseline to week 15 in mean total Patient-Reported Outcomes Measurement Information System (PROMIS)-29 domain scores; Patient Global Impression of Change (PGI-C) status at week 15; and change and percentage change from baseline to week 15 in mean total CRPS Severity Score (CSS).

Safety-related endpoints included the incidence of treatment-emergent adverse events (TEAEs), clinical laboratory test results, vital signs, Columbia-Suicide Severity Rating Scale (C-SSRS), and electrocardiographic parameters. Based on preclinical findings in rats, cataracts were considered an adverse event of special interest. Any participants with cataracts associated with visual acuity changes were immediately withdrawn from the study and were carefully monitored.

Exploratory endpoints included responder rate (defined as ≥ 30% improvement from baseline in the 24-hour pain intensity NPS score) during part B, and plasma 24HC levels at baseline and weeks 2, 7, and 29.

### Procedures

Pain intensity scores using the 24-hour 11-point NPS were collected daily using an E-diary during parts A and B. Participants were required to record their pain intensity scores for a minimum of 6 of 7 days before screening/enrollment, and then three times per day throughout the study. Pain intensity was evaluated on the affected limb. If more than one limb was involved, the participant and investigator determined which limb was the most affected and pain intensity was evaluated for that limb throughout the study. E-diaries had time-limited response windows and were available to the investigators in real time. Investigators were asked to call participants if they did not complete their daily E-diary.

The PROMIS-29 (version 2.1), PGI-C, and CSS were completed at screening, and at weeks 15 and 29. The PROMIS-29 is a generic health-related quality-of-life survey with seven domains: physical function, anxiety, depression, fatigue, sleep disturbance, ability to participate in social roles and activities, and pain interference. Answers were ranked by the participant on a 5-point Likert scale. Total raw domain scores were converted into T-scores using a conversion table based on data from a reference population [23]. The PGI-C involved answering the following question on a 7-point Likert scale (ranging from very much improved to very much worse): “Since beginning treatment at this clinic, how would you describe any changes (if any) in activity, limitations, symptoms, emotions and overall QoL related to your painful condition compared to before treatment?” The 16-point CSS was completed by clinicians based on the presence or absence of eight signs and eight symptoms of CRPS, including continuing disproportionate pain, allodynia, and hyperalgesia [24].

Blood samples (two 4 mL samples) to determine plasma 24HC levels were collected on day 1 (pre-dose and 1 hour post-dose) and weeks 2, 7, and 29 (1 hour post-dose). Adverse events were collected throughout the study until early termination or last follow-up visit (week 29), and the C-SSRS was assessed at baseline and weeks 1, 2, 3, 7, 11, and 15.

### Statistical analysis

Assuming a standard deviation (SD) of 2 and a 12% drop-out rate, a sample size of 24 participants (with a randomization ratio of 2:1 for soticlestat: placebo) was judged to be sufficient to achieve at least 65% power to detect a 2-point difference between soticlestat and placebo on the change from baseline to week 15 in mean 24-hour pain intensity NPS score at a two-sided significance level (two-sample *t*-test) of 0.10. A 2-point difference is within the accepted range of minimal clinically important difference for the 11-point NPS [25, 26].

Efficacy analyses were based on the full analysis set, which included all participants who were randomized, received at least one dose of the study drug, and had at least one valid post-baseline value for the assessment of mean 24-hour pain intensity score in part A or part B. The safety analysis set included all participants who received at least one dose of the study drug.

Observed values, change from baseline, and percentage change from baseline were summarized with descriptive statistics. Throughout the study, “baseline” was defined as the baseline in part A. For the primary efficacy endpoint, the average 24-hour pain intensity was calculated as the mean of three measurements collected by E-diary during 1 day. Data from the last 7 days before screening, week 15 (or last dose in part A, whichever was earlier), and week 29 (or last dose in part B, whichever was earlier) were used to derive the mean 24-hour pain intensity scores for baseline, week 15, and week 29, respectively. Linear mixed models for repeated measurements were used to evaluate the effect of soticlestat on mean 24-hour pain intensity in part A. Estimates for least-square means, least-square mean differences, 95% confidence intervals (CIs), and *P* values were obtained using the model with fixed effects for baseline NPS, site, visit, treatment, and treatment-by-visit interaction. An unstructured covariance matrix was assumed.

Statistical analyses were performed using SAS^®^ software (version 9.4; SAS Institute, Inc., Cary, NC, USA).

## Results

### Participants

In total, 24 participants were randomized and received treatment during part A (soticlestat, n = 15; placebo, n = 9) (Figure 1B). Of these, 18 participants completed part A (soticlestat, n = 12; placebo, n = 6) and enrolled in part B.

Baseline demographics were generally balanced between the soticlestat and placebo groups (Table 1). The median age of participants was 44.5 years (range, 18–62 years) and most were female (70.8%). Baseline pain intensity scores were similar between the soticlestat and placebo groups, with mean weekly averaged daily NPS pain intensity scores (SD) of 6.3 (1.0) and 6.3 (0.9), respectively. However, some differences were observed in baseline clinical characteristics, with the soticlestat group having a median time since symptom onset of 11.3 years (range, 2.6–20.5 years) vs 6.7 years (range, 1.4–7.4 years) in the placebo group. More participants in the soticlestat group were taking at least one opioid medication compared with participants in the placebo group (73.3% vs 33.3%, respectively). Additionally, concurrent medical conditions differed between the soticlestat and placebo groups (Table S1).

**Table 1.** Baseline demographics and clinical characteristics (safety analysis set)

	Soticlestat	Placebo	
	(n = 15)	(n = 9)	
Age, years			
Median (range)	46 (18–57)	38 (20–62)	
Sex, n (%)			
Male	4 (26.7)	3 (33.3)	
Female	11 (73.3)	6 (66.7)	
Race, n (%)			
White	15 (100.0)	9 (100.0)	
Distribution of CRPS to other limbs, n (%)	6 (40.0)	3 (33.3)	
Interruption of employment, education, or voluntary work since symptom onset, n (%)	14 (93.3)	9 (100.0)	
Dominant limb, n (%)			
Left	2 (13.3)	1 (11.1)	
Right	13 (86.7)	8 (88.9)	
First limb extremity affected by CRPS, n (%)			
Upper left	2 (13.3)	1 (11.1)	
Upper right	1 (6.7)	3 (33.3)	
Lower left	7 (46.7)	3 (33.3)	
Lower right	5 (33.3)	2 (22.2)	
Years since symptom onset			
Median (range)	11.3 (2.6–20.5)^b^	6.7 (1.4–7.4)^a^	
Years since diagnosis			
Median (range)	6.4 (0.3–13.9)^d^	3.6 (1.0–6.5)^c^	
Weekly averaged daily NPS pain intensity score, mean (SD)	6.3 (1.0)	6.3 (0.9)	
PROMIS-29, mean (SD)			
Physical function	34.7 (4.7)	35.8 (5.6)	
Anxiety	51.9 (8.9)	46.3 (11.9)	
Depression	47.3 (8.9)	47.3 (12.5)	
Fatigue	60.6 (8.3)	57.2 (8.4)	
Sleep disturbance	55.0 (2.4)	54.6 (3.9)	
Ability to participate in social roles and activities	39.1 (4.7)	40.0 (6.2)	
Pain interference	64.2 (8.0)	66.4 (6.7)	
CSS total score, mean (SD)	12.9 (1.9)	12.7 (1.7)	
At least one concomitant opioid medication, n (%)	11 (73.3)	3 (33.3)	
Concomitant pain medications, n (%)			
Amitriptyline	2 (13.3)	2 (22.2)	
Codeine	1 (6.7)	0 (0)	
Codeine/paracetamol	6 (40.0)	3 (33.3)	
Diclofenac	3 (20.0)	1 (11.1)	
Dihydrocodeine	1 (6.7)	1 (11.1)	
Fentanyl	2 (13.3)	0 (0)	
Ibuprofen	8 (53.3)	0 (0)	
Morphine	3 (20.0)	1 (11.1)	
Naproxen	3 (20.0)	2 (22.2)	
Paracetamol	5 (33.3)	5 (55.6)	
Tramadol	4 (26.7)	1 (11.1)	

CRPS = complex regional pain syndrome; CSS = CRPS Severity Score; NPS = Numeric Pain Scale; PROMIS = Patient-Reported Outcomes Measurement Information System; SD = standard deviation.

a n = 5.

b n = 6.

c n = 7.

d n = 8.

The median duration of treatment during part A was 103 days (range, 14–111 days) in the soticlestat group and 105 days (range, 7–108 days) in the placebo group. The median duration of treatment with soticlestat during part B was 98 days (range, 6–109 days).

During screening, all participants had an E-diary response rate of more than 90% for pain intensity scores. During treatment, only one participant out of 24 had a response rate of less than 90% (88.9%).

### Efficacy

**Figure 2.**
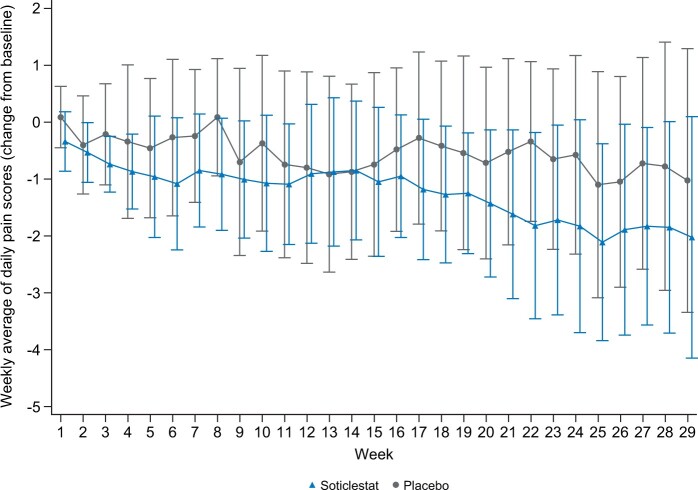
Plot of mean change from baseline in weekly averaged daily NPS scores during part A and part B (full analysis set). Data are mean ± SD. NPS = Numeric Pain Scale; SD = standard deviation.

For the primary efficacy endpoint, the soticlestat group did not differ from the placebo group, with least-square mean changes from baseline in the 24-hour pain intensity NPS score (95% CI) at week 15 of –0.75 (–1.55, 0.05) and –0.41 (–1.41, 0.59), respectively, resulting in a statistically non-significant placebo-adjusted difference of –0.34 (–1.55, 0.88; *P *=* *.570) (Table 2 and Figure 2). In addition, soticlestat treatment was associated with a statistically non-significant placebo-adjusted mean percentage difference in 24-hour pain intensity NPS score from baseline to week 15 of –3.26% (–20.11, 13.58; *P *=* *.703) (Table 2).

**Table 2.** Efficacy summary

Endpoint	Week 15 (part A)				Week 29 (part B)
	Soticlestat	Placebo	Difference		Soticlestat
	(n = 15)	(n = 9)	(95% CI)	*P* value^a^	(n = 18)
Change in 24-hour pain intensity NPS score from baseline, LS mean (95% CI)	–0.75 (–1.55, 0.05)	–0.41 (–1.41, 0.59)	–0.34 (–1.55, 0.88)	.570	–
Percentage change in 24-hour pain intensity NPS score from baseline, LS mean (95% CI)	–11.80 (–25.05, 1.45)	–8.54 (–23.17, 6.10)	–3.26 (–20.11, 13.58)	.703	–
Responders, n (%)^b^	4 (26.7)	2 (22.2)	–	–	6/24 (25.0)
Change in CSS total score from baseline,^c^ mean (SD)	–3.1 (3.12)	–2.2 (2.48)	–	.540	–4.4 (3.8)
Change in PROMIS-29 T-score from baseline,^d^ mean (SD)					
Physical function	2.4 (4.0)	1.5 (2.2)	–	–	3.8 (5.4)
Anxiety	–2.0 (9.6)	3.3 (10.3)	–	–	–3.9 (11.7)
Depression	–0.8 (6.9)	1.2 (10.0)	–	–	–0.5 (12.1)
Fatigue	–3.7 (10.5)	0.5 (12.1)	–	–	–2.4 (12.6)
Sleep disturbance	2.6 (1.9)	2.1 (5.1)	–	–	2.3 (3.7)
Ability to participate in social roles and activities	2.7 (5.2)	1.7 (6.1)	–	–	6.5 (8.9)
Pain interference	–0.4 (7.6)	–1.5 (6.3)	–	–	–6.8 (11.2)
PGI-C status, n (%)					
Very much improved	0 (0)	0 (0)	–	–	4 (22.2)
Much improved	5 (33.3)	3 (33.3)	–	–	6 (33.3)
Minimally improved	2 (13.3)	1 (11.1)	–	–	2 (11.1)
No change	5 (33.3)	2 (22.2)	–	–	2 (11.1)
Minimally worse	0 (0)	1 (11.1)	–	–	0 (0)

CI = confidence interval; CRPS = complex regional pain syndrome; CSS = CRPS Severity Score; LS = least-square; NPS = Numeric Pain Scale; PGI-C = Patients’ Global Impression of Change; PROMIS = Patient-Reported Outcomes Measurement Information System; SD = standard deviation.

– indicates no data is available.

a *P* values are provided for statistical analyses predefined in the protocol and/or that the study was powered for.

b Defined as ≥ 30% improvement from baseline on the 24-hour pain intensity NPS (n = 24).

c Higher scores indicate greater CRPS severity, that is, more signs/symptoms (range, 0–16 points) (n = 14).

^d^ Higher scores indicate more symptoms or function. For example, higher scores on the physical function scale represent better function, whereas higher scores on the depression scale indicate more depressive symptoms. Mean PROMIS-29 scores by visit are summarized in Table S2.

At week 15, four participants (26.7%) in the soticlestat group and two participants (22.2%) in the placebo group were classified as responders (≥ 30% improvement on the 24-hour pain intensity NPS). At week 29, six participants (25.0%) were classified as responders, comprising two participants (22.2%) who had received placebo and four (26.7%) who had received soticlestat during part A (Table 2).

Change from baseline to week 15 in mean CSS total score (SD) was –3.1 (3.12) points with soticlestat and –2.2 (2.48) points with placebo (*P *=* *.540) (Table 2). This reduction was maintained throughout part B, with a change from baseline to week 29 in mean CSS total score of –4.4 (3.8) points.

Minor changes were observed from baseline to week 15 in each of the seven PROMIS-29 domains, which were generally similar between the soticlestat group and the placebo groups (Table 2). Small improvements from baseline to week 15 were seen in both groups for physical function (soticlestat 2.4 [4.0]; placebo, 1.5 [2.2]), ability to participate in social roles and activities (soticlestat 2.7 [5.2]; placebo, 1.7 [6.1]), and pain interference (soticlestat –0.4 [7.6]; placebo, –1.5 [6.3]). Sleep disturbance became slightly worse in both groups (soticlestat 2.6 [1.9]; placebo, 2.1 [5.1]). Anxiety, depression, and fatigue showed small improvements in the soticlestat group (–2.0 [9.6], –0.8 [6.9], and –3.7 [10.5], respectively) but worsened slightly in the placebo group (3.3 [10.3], 1.2 [10.0], and 0.5 [12.1], respectively). Scores at week 15 were generally maintained to week 29 (Table 2). Mean PROMIS-29 scores by visit are summarized in Table S2.

An improvement in PGI-C status (much improved or minimally improved) from baseline to week 15 was reported by seven participants (46.7%) in the soticlestat group and four participants (44.4%) in the placebo group. At week 29, 12 participants (66.7%) reported improvement (very much, much, or minimally improved) (Table 2).

### 24HC levels

**Figure 3.**
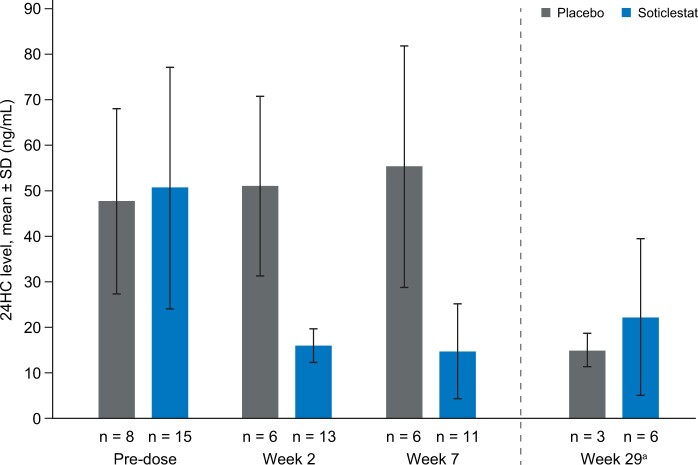
Mean plasma 24HC levels (pharmacodynamic analysis set). 24HC = 24S-hydroxycholesterol; SD = standard deviation. One participant who received soticlestat 100 mg and had baseline measurement only has not been included. ^a^All participants received soticlestat at week 29 (open-label part B); treatment during part A is indicated.

In participants receiving soticlestat, mean plasma 24HC levels decreased by approximately 70% in the first 2 weeks of treatment and remained at reduced levels throughout study parts A and B (Figure 3). Conversely, in participants receiving placebo, 24HC levels remained similar to those recorded at baseline at all available time points (Figure 3).

### Safety

During part A, 14 participants (93.3%) in the soticlestat group and 9 participants (100.0%) in the placebo group reported at least one TEAE, with TEAEs considered to be related to the study drug by the investigator in 8 (53.3%) and 6 (66.7%) participants, respectively (Table 3). During part B, 15 participants (83.3%) reported at least one TEAE; these events were considered to be related to the study drug by the investigator in five participants (27.8%). The most frequently observed TEAEs with soticlestat (in parts A and B) were headache (part A, n = 2 [13.3%]; part B, n = 5 [27.8%]), dizziness (part A, n = 4 [26.7%]), and nausea (part A, n = 3 [20.0%]; part B, n = 1 [5.6%]). Throughout the study, most TEAEs were mild or moderate. One severe TEAE occurred in one participant receiving soticlestat during part A (cholecystitis; considered unrelated to treatment) and two severe TEAEs occurred in one participant during part B (depression and suicidal ideation; considered related to treatment and leading to discontinuation). This second had a history of depression and reported a TEAE of depressed mood during Part A when receiving placebo, which ended the day before the TEAEs of depression and suicidal ideation were reported. Overall, three participants receiving soticlestat reported serious TEAEs (all during part A), none of which were considered by the investigator to be related to the study drug. These included the severe TEAE of cholecystitis described above, in addition to two moderate TEAEs of abdominal pain. In addition to the participant who discontinued during part B because of depression and suicidal ideation, one participant discontinued during part A (the individual who experienced the serious TEAE of cholecystitis) because of abnormal liver function test results, which turned out to be an early indication of progressive cholecystitis. No deaths were reported.

**Table 3.** Safety summary (safety analysis set)

	Part A		Part B
	Soticlestat	Placebo	Soticlestat
	(n = 15)	(n = 9)	(n = 18)
Participants experiencing at least one TEAE	14 (93.3)	9 (100.0)	15 (83.3)
Drug-related^a^	8 (53.3)	6 (66.7)	5 (27.8)
Mild	8 (53.3)	7 (77.8)	8 (44.4)
Moderate	5 (33.3)	2 (22.2)	6 (33.3)
Severe	1 (6.7)	0 (0)	1 (5.6)
Leading to drug discontinuation	1 (6.7)	0 (0)	1 (5.6)
Adverse events of special interest^b^	0 (0)	0 (0)	3 (16.7)
Participants experiencing at least one serious TEAE	3 (20.0)	0 (0)	0 (0)
Drug-related	0 (0)	0 (0)	0 (0)
TEAEs reported by ≥ 15% of participants in any group			
Headache	2 (13.3)	4 (44.4)	5 (27.8)
Dizziness	4 (26.7)	2 (22.2)	0 (0)
Cough	1 (16.7)	3 (33.3)	1 (5.6)
Nausea	3 (20.0)	1 (11.1)	1 (5.6)
Nasopharyngitis	1 (6.7)	2 (22.2)	1 (5.6)
Depressed mood	0 (0)	2 (22.2)	1 (5.6)

TEAE = treatment-emergent adverse event.

Data are expressed as n (%).

a TEAEs considered to be related to treatment in Part A: abdominal pain (placebo, n = 1); abnormal dreams (soticlestat, n = 1); anxiety (placebo, n = 1); constipation (soticlestat, n = 1); disturbance in attention (placebo, n = 1); dizziness (soticlestat, n = 4; placebo, n = 2); dry mouth (soticlestat, n = 2); dyspepsia (soticlestat, n = 1); eosinophil count increased (soticlestat, n = 1); fatigue (soticlestat, n = 2); headache (soticlestat, n = 2; placebo, n = 4); insomnia (soticlestat, n = 2; placebo, n = 1); lethargy (soticlestat, n = 1; placebo, n = 1); nausea (soticlestat, n = 3; placebo, n = 1); and visual hallucination (placebo, n = 1). TEAEs considered to be related to treatment in Part B: atrioventricular block first degree (n = 1); cataract (n = 1); cataract cortical (n = 1); cataract subcapsular (n = 1); depression (n = 1); drug withdrawal syndrome (n = 1); headache (n = 2); insomnia (n = 1); and suicidal ideation (n = 1). Participants may have reported more than one TEAE.

b Psychosis, convulsions, and cataracts.

Three participants (5.6%) were found to have mild cataracts: one participant who received placebo during part A was found to have lesions before starting soticlestat treatment in part B; one participant who received soticlestat during part A and part B had an ongoing mild lesion, thought to be a borderline age-related cataract; and one participant who received soticlestat during part A and part B had lesions reported at week 13 that were considered not significant and were likely related to age. None of the lesions affected visual acuity in any of the participants and none were considered to be clinically significant.

A maximum post-baseline category of suicidal ideation according to the C-SSRS was recorded in two participants (11.1%) during part B.

## Discussion

In this proof-of-concept phase 2a study, soticlestat, a CH24H inhibitor, was investigated as adjunctive therapy in participants with chronic CRPS. The study did not show significance for its primary endpoint, with no statistically significant difference in mean 24-hour pain intensity NPS score between soticlestat and placebo groups observed after 15 weeks of treatment. Similar rates of TEAEs were reported in the soticlestat and placebo groups, and most were mild or moderate.

Small improvements were noted in some PROMIS-29 domains at week 15 in both the soticlestat and placebo groups, with several T-score improvements at week 29 falling within the minimal important change range of 2–6 T-score points proposed for PROMIS-29 measures when evaluating non-surgical interventions [27]. For example, at the end of Part B, the change from baseline in T-scores for ability to participate in social roles and activities, and pain interference were greater than 6. However, given the trend toward improvement in the placebo arm during part A and lack of statistical analyses owing to the study design, it is impossible to say whether this is an effect of soticlestat treatment. Similarly, the improvements noted in the PGI-C at week 29 must be interpreted with caution because they cannot be disentangled from the possible placebo effects associated with being in a clinical study.

Sustained reductions in plasma 24HC levels were seen in participants receiving soticlestat, whereas 24HC levels in participants receiving placebo remained similar to baseline values. This indicates that participants adhered to treatment and supports the use of 24HC as a biomarker for soticlestat activity. Decreases in plasma 24HC levels observed in the present study were similar to those seen in other soticlestat clinical studies [21, 22, 28, 29].

CRPS is a multi-factorial condition with many mechanisms that may change the course of the disease [8]. People with CRPS report chronic pain, multiple system dysfunction, and loss of QoL [4, 30]. Despite an interdisciplinary approach to treatment—which usually includes functional restoration, pharmacological therapies, interventional procedures, and psychological interventions 8, 31]—the management of pain is often suboptimal [4]. The costs associated with CRPS are significant, both economically and in terms of quality of life [7, 32]. Despite a clear need for effective treatments, scant evidence exists to guide treatment and few medications that are used clinically have been tested rigorously [8].

The NMDA receptor antagonist ketamine may provide clinically effective short-term pain relief in CRPS [15]. Indeed, a meta-analysis indicated that ketamine infusion can provide effective pain relief for up to 3 months [15]. In light of this, soticlestat was hypothesized to decrease pain via its indirect NMDA modulation, with reduced 24HC levels leading to a reduction in glutamatergic activation and inflammatory responses. In this study, the reduction in 24-hour average pain intensity resulting from soticlestat treatment fell short of the predefined 2-point target deemed to be clinically meaningful [33]. One possible explanation for the lack of efficacy observed in the present study could be the duration of CRPS in the included population. Given the pathophysiological role of neurogenic inflammation following the initial trauma, the likelihood of a response to soticlestat may be greater in individuals at the onset of CRPS [12, 34]. Although small improvements in some efficacy measures, including those relating to global functioning, were maintained or enhanced with longer treatment, any potential treatment effect throughout part B could not be assessed statistically because of the lack of a control arm.

Overall, the safety findings of soticlestat treatment in this study were consistent with those from previous clinical studies [21, 22, 28], and no new safety signals emerged in participants with CRPS. Most TEAEs were mild or moderate, and none of the serious TEAEs reported with soticlestat treatment were considered by the investigators to be related to the study drug. One severe event was reported (depression and suicidal ideation), which was considered related to soticlestat treatment and led to discontinuation. Of note, suicidal ideation has not been reported in any other soticlestat clinical study, including an estimated 533 individuals who have received soticlestat (as of May 28, 2022). The most frequently observed TEAEs with soticlestat in the present study were headache, dizziness, and nausea.

Study strengths include the use of the Budapest Criteria, which is recognized as the international standard for CRPS diagnosis [8, 35], with all participants having experienced CRPS symptoms for at least 6 months before study entry. The Budapest Criteria were designed to be used as general clinical diagnostic criteria; it includes most of the signs and symptoms of CRPS, and it is therefore subjective and quasi-objective (physical examination). The inclusion of a control arm allowed for statistical analysis of the effects of soticlestat vs placebo. The study also included a range of outcome measures covering aspects of health and daily functioning considered to be important by patients, all of which are recognized as core outcome measures for CRPS [36].

A key limitation of this study is its small sample size, which limited the statistical analyses. Although the number of participants specified in the protocol were recruited, the drop-out rate was higher than expected and further recruitment was affected by the coronavirus disease 2019 (COVID-19) pandemic. Furthermore, the study size was planned to achieve at least 65% power, which may have been too low to detect a true effect. Inferential statistical analyses were only undertaken for outcomes predefined in the protocol and as per the power calculation for the study. Additionally, despite randomization, there was an imbalance in key factors between treatment arms at baseline. Relative to the placebo group, the soticlestat group had experienced a longer mean time since injury, symptom onset, and diagnosis. They also had more nervous system and immune disorders than the placebo group. Furthermore, participants in the soticlestat group were more likely to be taking opioid medication than participants in the placebo group. Given the possibility of opioid-induced hyperalgesia [37], this factor should be considered in any future study design. These differences, along with the potential inclusion of treatment-resistant participants, represent potential confounding factors in data interpretation. The present study design did not allow for analysis of any potential cumulative effect of treatment over the entire study period.

In conclusion, soticlestat as adjunctive therapy did not reduce pain intensity in participants with chronic CRPS. Small improvements during Part A of the study in both the treatment and placebo groups were maintained or further improved upon following extended treatment with soticlestat. Overall, however, the small sample size and the imbalance between treatment groups limited the interpretation of the findings from this study.

## Supplementary Material

pnac198_Supplementary_Data

## Data Availability

The datasets, including the redacted study protocol, redacted statistical analysis plan, and individual participants data supporting the results reported in this article, will be available three months after the submission of a request, to researchers who provide a methodologically sound proposal. The data will be provided after its de-identification, in compliance with applicable privacy laws, data protection and requirements for consent and anonymization.

## Notes

**ClinicalTrials.gov identifier:** NCT03990649.

**Date patient enrolment began:** July 23, 2019.
