# Supplementary material for: Randomized controlled study to evaluate the efficacy and safety of soticlestat as adjunctive therapy in adults with complex regional pain syndrome
Source: Pain Med. 2022 Dec 20;24(7):872–80. doi: 10.1093/pm/pnac198 (PMC10321763; doi:10.1093/pm/pnac198)
Supplement: pnac198_Supplementary_Data [file pnac198_supplementary_data.docx]

# Supplementary material

**Supplementary Table 1.** Concurrent medical conditions by system organ class

|  | **Part A** | |  |
| --- | --- | --- | --- |
|  | **Soticlestat**  **(n = 15)** | **Placebo**  **(n = 9)** |  |
| Congenital, familial, and genetic disorders | 3 (20.0) | 1 (11.1) |  |
| Ear and labyrinth disorders | 1 (6.7) | 0 |  |
| Endocrine disorders | 1 (6.7) | 0 |  |
| Eye disorders | 0 | 1 (11.1) |  |
| Gastrointestinal disorders | 2 (13.3) | 3 (33.3) |  |
| General disorders and administration site conditions | 1 (6.7) | 0 |  |
| Immune system disorders | 7 (46.7) | 1 (11.1) |  |
| Infections and infestations | 2 (13.3) | 0 |  |
| Injury, poisoning, and procedural complications | 1 (6.7) | 0 |  |
| Investigations | 2 (13.3) | 0 |  |
| Metabolism and nutrition disorders | 8 (53.3) | 4 (44.4) |  |
| Musculoskeletal and connective tissue disorders | 7 (46.7) | 3 (33.3) |  |
| Neoplasms benign, malignant, and unspecified (incl. cysts and polyps) | 0 | 2 (22.2) |  |
| Nervous system disorders | 3 (20.0) | 0 |  |
| Product issues | 1 (6.7) | 0 |  |
| Psychiatric disorders | 7 (46.7) | 4 (44.4) |  |
| Renal and urinary disorders | 1 (6.7) | 2 (22.2) |  |
| Reproductive system and breast disorders | 1 (6.7) | 2 (22.2) |  |
| Respiratory, thoracic, and mediastinal disorders | 4 (26.7) | 2 (22.2) |  |
| Skin and subcutaneous tissue disorders | 6 (40.0) | 1 (11.1) |  |
| Social circumstances | 1 (6.7) | 1 (11.1) |  |
| Surgical and medical procedures | 0 | 1 (11.1) |  |
| Vascular disorders | 2 (13.3) | 1 (11.1) |  |

All data are presented as n (%).

**Supplementary Table 2.** Summary PROMIS-29 scores by domain and visit

|  | **Baseline** | |  | | **Part A** | | | |  | | **Part B** | | | |  |
| --- | --- | --- | --- | --- | --- | --- | --- | --- | --- | --- | --- | --- | --- | --- | --- |
|  | **Soticlestat** | **Placebo** |  | | **Soticlestat** | | **Placebo** | |  | | **Soticlestat** | | **Placebo** | |  |
| Physical function |  |  |  | |  | |  | |  | |  | |  | |  |
| n | 15 | 9 |  | | 12 | | 8 | |  | | 12 | | 6 | |  |
| Score, mean (SD) | 34.65 (4.743) | 35.83 (5.607) |  | | 36.38 (4.965) | | 38.51 (4.914) | |  | | 39.61 (7.599) | | 39.20 (3.762) | |  |
| Anxiety |  |  |  | |  | |  | |  | |  | |  | |  |
| n | 15 | 9 |  | | 13 | | 8 | |  | | 12 | | 6 | |  |
| Score, mean (SD) | 51.94 (8.873) | 46.30 (11.906) |  | | 49.87 (9.317) | | 46.96 (9.939) | |  | | 44.58 (7.646) | | 47.03 (11.283) | |  |
| Depression |  |  |  | |  | |  | |  | |  | |  | |  |
| n | 15 | 9 |  | | 13 | | 8 | |  | | 12 | | 6 | |  |
| Score, mean (SD) | 47.27 (8.868) | 47.31 (12.523) |  | | 46.62 (6.181) | | 45.70 (8.974) | |  | | 46.81 (7.917) | | 46.38 (13.186) | |  |
| Fatigue |  |  |  | |  | |  | |  | |  | |  | |  |
| n | 15 | 9 |  | | 13 | | 8 | |  | | 12 | | 6 | |  |
| Score, mean (SD) | 60.55 (8.280) | 57.17 (8.427) |  | | 56.57 (8.539) | | 56.93 (10.308) | |  | | 55.59 (8.629) | | 56.60 (12.355) | |  |
| Sleep disturbance |  |  |  | |  | |  | |  | |  | |  | |  |
| n | 15 | 9 |  | | 13 | | 8 | |  | | 12 | | 6 | |  |
| Score, mean (SD) | 54.99 (2.390) | 54.63 (3.854) |  | | 57.52 (2.019) | | 56.58 (2.536) | |  | | 57.18 (2.537) | | 55.82 (2.141) | |  |
| Ability to participate in social roles and activities | | | |  | |  | |  | |  | |  | |  | |
| n | 15 | 9 |  | | 13 | | 7 | |  | | 12 | | 6 | |  |
| Score, mean (SD) | 39.11 (4.723) | 40.03 (6.205) |  | | 41.63 (6.845) | | 43.41 (6.691) | |  | | 46.66 (9.309) | | 45.93 (10.628) | |  |
| Pain interference |  |  |  | |  | |  | |  | |  | |  | |  |
| n | 15 | 9 |  | | 13 | | 7 | |  | | 12 | | 6 | |  |
| Score, mean (SD) | 64.22 (8.039) | 66.37 (6.710) |  | | 63.58 (6.500) | | 63.04 (5.242) | |  | | 55.13 (11.453) | | 60.83 (7.586) | |  |

PROMIS = Patient-Reported Outcomes Measurement Information System; SD = standard deviation.

PROMIS-29 includes six domains with four questions plus one domain ranking numeric pain score (0–10). When all questions are answered, the total raw score for each domain is calculated by summing all the scores in the corresponding domain. The total raw score is then converted to a T-score for each domain. Higher scores indicate more symptoms or function. For example, higher scores on the physical function scale represent better function, whereas higher scores on the depression scale indicate more depressive symptoms.

Participants who completed Part B are summarized by treatment received in Part A.
